# PAWS1 controls cytoskeletal dynamics and cell migration through association with the SH3 adaptor CD2AP

**DOI:** 10.1242/jcs.202390

**Published:** 2018-01-01

**Authors:** Timothy D. Cummins, Kevin Z. L. Wu, Polyxeni Bozatzi, Kevin S. Dingwell, Thomas J. Macartney, Nicola T. Wood, Joby Varghese, Robert Gourlay, David G. Campbell, Alan Prescott, Eric Griffis, James C. Smith, Gopal P. Sapkota

**Affiliations:** 1 Medical Research Council Protein Phosphorylation and Ubiquitylation Unit, Dundee DD1 5EH, UK; 2 The Francis Crick Institute, 1 Midland Road, London NW1 1AT, UK; 3 Cell Signalling and Immunology, University of Dundee, Dundee DD1 5EH, UK; 4 Centre for Gene Regulation and Expression, University of Dundee, Dundee DD1 5EH, UK

**Keywords:** PAWS1, FAM83G, CD2AP, Cell migration, Actin cytoskeleton

## Abstract

Our previous studies of PAWS1 (protein associated with SMAD1; also known as FAM83G) have suggested that this molecule has roles beyond BMP signalling. To investigate these roles, we have used CRISPR/Cas9 to generate PAWS1-knockout U2OS osteosarcoma cells. Here, we show that PAWS1 plays a role in the regulation of the cytoskeletal machinery, including actin and focal adhesion dynamics, and cell migration. Confocal microscopy and live cell imaging of actin in U2OS cells indicate that PAWS1 is also involved in cytoskeletal dynamics and organization. Loss of PAWS1 causes severe defects in F-actin organization and distribution as well as in lamellipodial organization, resulting in impaired cell migration. PAWS1 interacts in a dynamic fashion with the actin/cytoskeletal regulator CD2AP at lamellae, suggesting that its association with CD2AP controls actin organization and cellular migration. Genetic ablation of CD2AP from U2OS cells instigates actin and cell migration defects reminiscent of those seen in PAWS1-knockout cells.

This article has an associated First Person interview with the first authors of the paper.

## Abstract (precis)

**Summary:** PAWS1 (also known as FAM83G) controls cell migration by influencing the organization of F-actin and focal adhesions and the distribution of the actin stress fibre network through its association with CD2AP.

## INTRODUCTION

Cell migration is involved in embryonic development, wound healing, the immune response and cancer metastasis (Easley et al., 2008; Fife et al., 2014). Although many of the molecules and biophysical processes involved in cell migration have been identified and characterized (Huber et al., 2015; Leduc and Etienne-Manneville, 2015; Mohapatra et al., 2016), we do not have a complete understanding of the process. One of the most important properties of cell migration is the ability of cells to fine-tune their cytoskeletal structure in response to changing environmental cues such as growth factor stimulation (Dang et al., 2013; Krause and Gautreau, 2014; Mendoza et al., 2015; Timpson et al., 2011).

Cytoskeletal components such as actin and tubulin play important roles in migration and invasion, notably in the pathology of tumour cells (Shortrede et al., 2016). Actin takes two forms: monomeric globular (G-actin) and filamentous (F-actin). F-actin polymerization is responsible for dynamic changes in cell shape and for chemotactic responses to growth factor signalling. It is also involved in the formation of lamellipodia, filopodia and other macromembrane structures that drive directional or chemotactic migration (Johnson et al., 2015; Kelley et al., 2010; King et al., 2016; Welf et al., 2012). Without properly regulated actin polymerization and branching, cells are unable to properly sense their microenvironment and they may display unregulated migratory behaviour.

The organization and polymerization of actin are controlled by molecular complexes that include actin-related protein 2/3 (Arp2/3) and Wiskott-Aldrich syndrome protein (WASP)/WASP-family verprolin-homologous protein (WAVE) regulators that are downstream of the small GTPases Rho, Rac and Cdc42 (Devreotes and Horwitz, 2015; Guo et al., 2006). Dynamic membrane structures such as invadopodia, lamellipodia and pseudopodia are formed through the regulation of actin polymerization through association with nucleators, crosslinkers, capping proteins, severing proteins, debranching proteins and myosin motors (Chi et al., 2014; Lehtimaki et al., 2016; Mierke, 2015). One such regulator is the adaptor protein CD2AP, which delivers capping proteins to the barbed ends of polymerizing F-actin. Capping growing filaments can promote the formation of actin branches by increasing the G-actin pool available to form branches (Akin and Mullins, 2008). The resulting change in network architecture leads to plasma membrane ruffling, chemotactic arching and, eventually, motility (Bruck et al., 2006; Tang and Brieher, 2013; Zhao et al., 2013). The branch-promoting activity of CD2AP, together with the action of the capping proteins CAPZA1 and CAPZB, leads to modifications in branched actin and causes membrane distortion and changes in tight junctions (Tang and Brieher, 2013; Zhao et al., 2013).

PAWS1 (also known as FAM83G) is a member of the FAM83 family of proteins that is characterized by the presence of a conserved DUF1669 domain of unknown function. The domain includes a pseudo-phospholipase D (PLD) catalytic motif, so-called because no PLD activity has been detected in FAM83 proteins (Cipriano et al., 2012, 2013). Outside the DUF1669 domain, the FAM83 members are distinct, perhaps pointing to different roles for each member. We have previously shown that PAWS1 interacts with SMAD1 and modulates bone morphogenetic protein (BMP) signalling and transcription (Vogt et al., 2014); here, we demonstrate that loss of PAWS1 causes profound morphological and migratory changes in cells. A proteomic screen of the FAM83 family of proteins reveals that in addition to the SMADs, PAWS1 interacts with CD2AP. Bearing in mind the key roles of CD2AP in cytoskeletal organization, dynamics and cell migration, this observation suggests that PAWS1 might interact with CD2AP to regulate cytoskeletal machinery and cell migration (Bruck et al., 2006; Tang and Brieher, 2013; Zhao et al., 2013). Our results indicate that PAWS1 is a novel regulator of actin-cytoskeletal dynamics, cell locomotion and migration. Knocking out PAWS1 from U2OS osteosarcoma cells causes actin cytoskeletal and cell migration defects similar to those caused by the loss of CD2AP, suggesting that the association between PAWS1 and CD2AP plays an important role in regulating cytoskeletal dynamics and cell migration.

## RESULTS

### PAWS1 deficiency affects cell morphology, cytoskeletal dynamics and migration

**Fig. 1.**
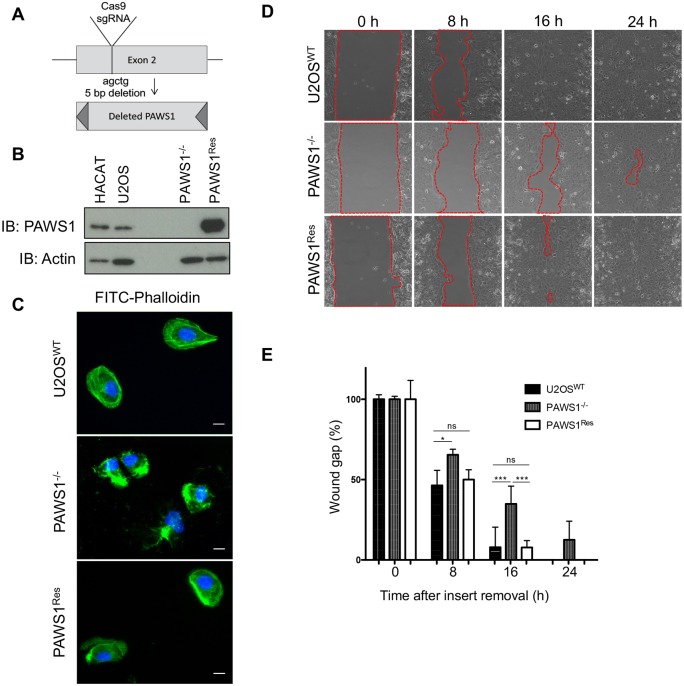
**Loss of PAWS1 elicits defects in U2OS cell migration and morphology.** (A) CRISPR-mediated deletion of PAWS1 at exon 2 of the PAWS1 gene. (B) Anti-PAWS1 immunoblots (IB) of 20 µg extracts from control HaCaT keratinocytes and U2OS osteosarcoma cells, as well as targeted PAWS1-knockout (PAWS1^−/−^) U2OS cells and knockout cells rescued with WT PAWS1 (PAWS1^Res^). (C) Fluorescence microscopy of actin [FITC–phalloidin (green)] and DAPI (blue) staining in WT control U2OS cells, PAWS1^−/−^ cells or PAWS1^Res^ cells depicting actin organization. Scale bars: 10 µm. (D) Time-lapse wound healing migration of WT (U2OS), PAWS1^−/−^ and PAWS1^Res^ cells at 0, 8, 16, and 24 h following removal of the insert separating wells of confluent cells. Images were taken under phase microscopy at 20× magnification. (E) The percentage of wound (gap) closure (as indicated in D) was quantified and plotted as shown (mean±s.d.; *n*=3). *P<0.0160, ***P=0.0007; ns, not significant (one-way ANOVA with Tukey's adjustment for multiple comparisons test).

To investigate the functions of PAWS1, we generated PAWS1-knockout U2OS cells (PAWS1^−/−^) by CRISPR/Cas9 targeting of exon 2 of the PAWS1 gene (Fig. 1A). The loss of PAWS1 protein in the isolated clone of U2OS cells was verified by western blotting (Fig. 1B), while genomic sequencing surrounding the *sgRNA* target site revealed a 5-base pair deletion from both alleles (Fig. 1A). For rescue experiments, we employed a previously described retroviral method (Vogt et al., 2014) to stably restore the expression of wild-type (WT) PAWS1 in PAWS1^−/−^ cells (PAWS1^Res^). We note that levels of PAWS1 in PAWS1^Res^ cells were substantially higher than the endogenous levels in control U2OS and HaCaT keratinocyte cells (Fig. 1B). Under these conditions, phalloidin staining of fixed PAWS1^−/−^ U2OS cells showed a disorganized and tangled mesh of actin, while WT U2OS cells and PAWS1^Res^ cells showed normal actin stress fibre organization (Fig. 1C). Inspection of actin fibre organization in PAWS1^−/−^ and WT U2OS cells revealed more filopodia-like or retraction fibre-like protrusions in PAWS1^−/−^ cells compared with those in the WT cells (Fig. S1A,B).

Abnormal actin organization and cell shape can cause defects in cell migration (Schratt et al., 2002; Zaoui et al., 2008). To assess the role of PAWS1 in cell migration, we performed a lateral wound-healing assay (Huang et al., 2009). WT, PAWS1^−/−^ and PAWS1^Res^ U2OS cells were cultured to confluency in adjacent chambers of a culture well divided by a small fixed-sized spacer, such that a uniform gap was created when the spacer was removed. Cell migration into the gap was monitored for up to 24 h (Fig. 1D). Fewer PAWS1^−/−^ cells had migrated into the gap than WT or PAWS1^Res^ U2OS cells at both 16 h and 24 h (Fig. 1D). After 16 h, PAWS1^−/−^ cells showed 60% wound closure relative to the starting wound gap, compared with ∼85% for WT and PAWS1^Res^ U2OS cells (Fig. 1E). In a similar assay, live imaging of PAWS1^−/−^ and WT U2OS cells on opposite sides of the wound showed that, while WT cells can form well-defined membrane ruffles and lamellipodia and migrate rapidly across the wound gap, PAWS1^−/−^ cells remain tightly connected to each other, form poorly defined membrane ruffles and lamellipodia and migrate slowly (Movie 1). We also investigated the migration over time of WT, PAWS1^−/−^ and PAWS1^Res^ cells towards a chemoattractant after seeding cells in serum-free conditions on µ-Slide chemotaxis chambers with 10% fetal bovine serum (FBS) added on adjacent chambers as chemoattractant. Although no significant differences in the directionality of cell migration towards FBS were observed over the course of this assay, PAWS1^−/−^ cells displayed a striking delay in adhesion compared to WT or PAWS1^Res^ cells (Fig. S1F,G). We note that overexpression of PAWS1 in WT U2OS cells also caused delayed migration into the wound (Fig. S1C–E). Collectively, these observations indicate that PAWS1 plays a role in actin organization, cell adhesion and cell migration in U2OS cells.

### Phenotypic characterization of PAWS1 actin defects

**Fig. 2.**
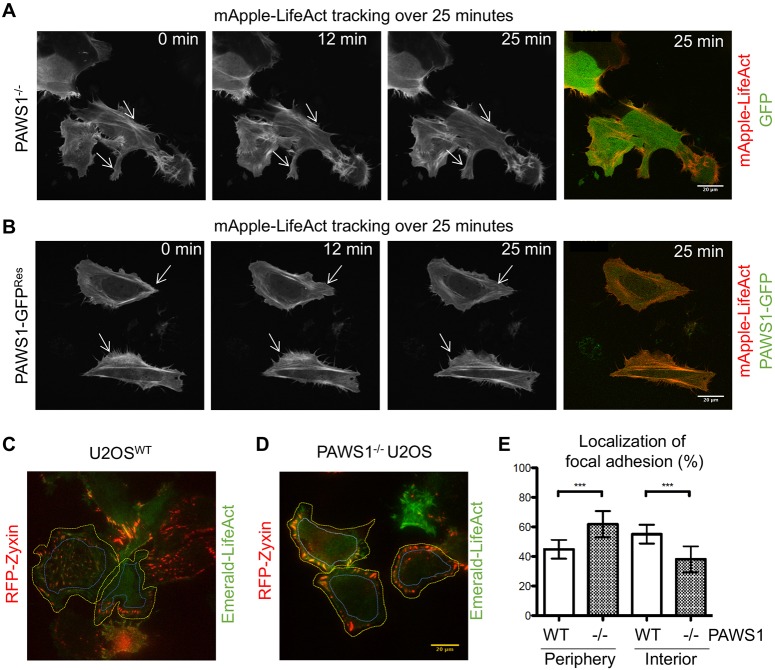
**The effect of PAWS1 on actin and focal adhesion dynamics and distribution.** (A) GFP and LifeAct–mApple were transfected into PAWS1^−/−^ U2OS cells, which were imaged for 25 min using a Zeiss confocal microscope at 60× magnification. Representative still images at the indicated times are presented. Static regions of membrane ruffles are indicated by the arrows. See Movie 2 for actin dynamics over the timecourse of 25 min. (B) As in A, except that PAWS1–GFP and mApple–LifeAct were transfected into PAWS1^−/−^ U2OS cells and imaged for 25 min. The dynamic ruffling of the membrane is indicated by the arrows. See Movie 3 for actin dynamics over the timecourse of 25 min. (C) U2OS WT or (D) PAWS1^−/−^ cells were transfected with RFP–Zyxin (punctate staining) and Emerald LifeAct, then imaged by TIRF microscopy at 60× magnification for 30 min to determine membrane dynamics of focal adhesions and cytoskeletal association. (E) The number of focal adhesions were quantified (RFP–Zyxin) with ImageJ at the cell periphery, as indicated by the yellow outer perimeter and the inner blue boundary. Interior adhesions were measured inside of the blue boundary. The number of focal adhesions in the periphery or interior were then expressed as relative to the total number of adhesions in the cell (as a percentage; mean±s.d.; *n*=3). ***P<0.001 (one-way ANOVA with Tukey's multiple comparisons test). Scale bars: 20 µm.

To understand how PAWS1 affects cytoskeletal dynamics, we first performed live-cell imaging of PAWS^−/−^ U2OS cells transfected with either GFP control or PAWS1–GFP together with mApple–LifeAct (Fig. 2A,B). PAWS1^−/−^ control cells displayed disorganized and static actin kinetics, suggesting that PAWS1 deletion causes defects in the organization and dynamics of the actin network (Fig. 2A; Movie 2). In contrast, cells transfected with PAWS1–GFP had an organized and dynamic actin network, and membrane ruffling was observed throughout the 25 min imaging period (Fig. 2B; Movie 3). Thus, the introduction of PAWS1–GFP in PAWS1^−/−^ cells was sufficient to restore membrane dynamics and the localization of actin in stress fibres (Fig. 2B; Movie 3).

Focal adhesions are anchors of cell protrusions towards the extracellular matrix. They organize the actin cytoskeleton and allow traction forces to be generated to move the cell body (Guo et al., 2006). We asked whether focal adhesions are also affected by loss of PAWS1. Live-cell total internal reflection fluorescence (TIRF) microscopy was carried out over 25 min on PAWS1^−/−^ U2OS or WT cells transfected with the focal adhesion protein RFP–zyxin to observe focal adhesion dynamics and distribution (Fig. 2C,D; Movies 4 and 5). In WT cells, zyxin displayed the expected punctate pattern throughout the basal surface of cells (Fig. 2C,E; Fig. S2). In contrast, PAWS1^−/−^ cells had a marked peripheral distribution of zyxin (Fig. 2D,E; Fig. S2), indicating that focal adhesions fail to form properly.

### Micropattern analysis of cytoskeletal actin fibres and cortactin in PAWS1^−/−^ U2OS cells

**Fig. 3.**
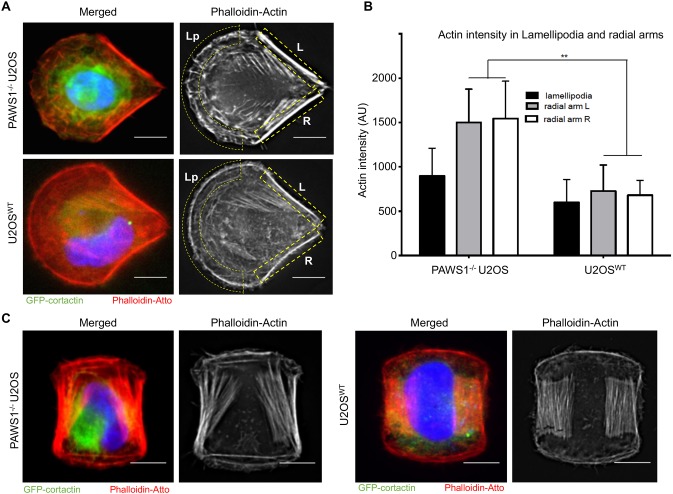
**Micropattern cyclic strain analysis of PAWS1^−/−^ U2OS and WT U2OS cells.** (A) PAWS1^−/−^ (upper panel) or WT U2OS cells (lower panel) transfected with GFP–cortactin for 24 h (as a secondary measure of membrane dynamics) and then stained with phalloidin–Atto and DAPI. Crossbow micropattern chips were coated with 20 µg/ml fibronectin and cells were seeded at low density to allow adhesion of ∼1 cell per pattern following gentle washing. Actin stress fibre organization was measured through wide-field deconvolution microscopy. GFP–cortactin is in green, phalloidin–actin is in red and DAPI is in blue. (B) Quantification of 10 (WT) and 15 (PAWS1^−/−^) images of each cell genotype was performed with ImageJ to determine the accumulation of actin in the lamellipodia (Lp in A) and radial ventral arms at the trailing edge of the cells (indicated by L and R in yellow dashed boxes in A). Arbirtrary intensity units were measured. **P<0.05 (Student's *t*-test). (C) As in A, except that double-crossbow H-patterned fibronectin-coated chips were used to plate PAWS1^−/−^ and WT U2OS cells. Scale bars: 20 µm.

Bearing in mind the role of PAWS1 in cell morphology, migration, cytoskeletal organization and focal adhesion distribution, we decided to examine its contribution to the architecture of cortactin and actin fibres. To this end, PAWS1^−/−^ and control U2OS cells were plated onto fibronectin-coated crossbow and H-shaped (double-crossbow) micropatterns (Versaevel et al., 2017). We first noted that the lamellipodia of PAWS1^−/−^ cells plated on the ‘crossbow’ fibronectin micropattern had a disorganized actin pattern (Fig. 3A; Fig. S3A). Thus, in control cells there was a clearly defined continuous belt of actin that spanned the leading adhesive edge. However, in PAWS1^−/−^ cells we noted that this band was discontinuous and there were several spike-like actin projections (Fig. 3A; Fig. S3A). Control and PAWS1^−/−^ cells both showed the expected accumulation of actin along non-adhesive edges (Théry et al., 2006), but stress fibres between the adhesive regions of PAWS1^−/−^ cells were brighter than those in control cells (Fig. 3A,B). In the double crossbow micropattern, in addition to the defects observed above, we noted that stress fibres between the adhesion arms were not organized into proper parallel arrays in the PAWS1^−/−^ cells (Fig. 3C; Fig. S3B). There were no substantial differences in the distribution of GFP–cortactin between WT and PAWS1^−/−^ cells in either micropattern, although the GFP–cortactin signal appeared to be more intense in PAWS1^−/−^ cells (Fig. 3C). While micropatterns are useful in visualizing actin distribution, we note that they do not represent true physiological states of cells but reflect forced and exaggerated actin structures.

### PAWS1 interacts with CD2AP, a key regulator of actin cytoskeleton

**Fig. 4.**
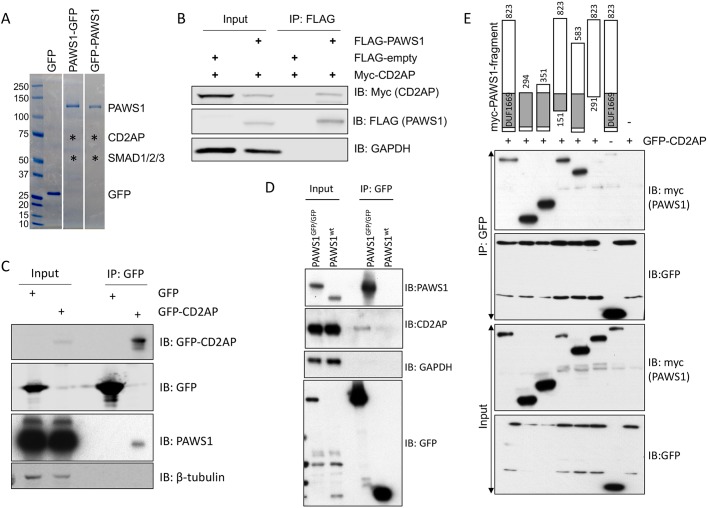
**PAWS1 interacts with CD2AP.** (A) Anti-GFP immunoprecipitations from extracts of HEK293 cells expressing GFP alone, or PAWS1 tagged with GFP either at the C- or the N-terminus, were resolved by SDS-PAGE, and interacting proteins identified by MS. The Coomassie stained gels indicating the approximate positions from where the designated interacting proteins were identified are included. (B) Verification of interactions between Myc-tagged CD2AP and Flag-tagged PAWS1 by co-expression and immunoprecipitation (IP) experiments performed in PAWS1^−/−^ U2OS cells as indicated. IB, immunoblot. (C) Anti-GFP immunoprecipitations from extracts of cells either expressing GFP or GFP–CD2AP were subjected to immunoblotting with anti-GFP or anti-PAWS1 antibodies as indicated. (D) Homozygous PAWS1–GFP-knockin U2OS cells (PAWS1^GFP/GFP^), in which the GFP tag was introduced at the C-terminus of PAWS1 gene on both allelles by using CRISPR/Cas9, and WT U2OS cells transfected with GFP control were subjected to anti-GFP immunoprecipitations. Extracts and anti-GFP immunoprecipitations were then subjected to immunoblotting with anti-PAWS1 and anti-CD2AP as indicated. (E) Mapping minimal PAWS1 region necessary for interaction with CD2AP. The indicated fragments of Myc-tagged PAWS1 were co-expressed with either GFP or GFP–CD2AP in PAWS1^−/−^ U2OS cells for 48 h. Extracts or anti-GFP immunoprecipitations were subjected to immunoblotting with anti-Myc and anti-GFP antibodies as indicated.

In order to understand the molecular mechanism by which PAWS1 modulates actin cytoskeletal organization, we used mass spectrometry (MS) to identify PAWS1 interactors from tetracycline-inducible HEK293 cells (Yao et al., 2007, 1998) stably integrated with a single copy of either N-terminally or C-terminally GFP-tagged PAWS1 or GFP alone as control (Fig. 4A). GFP-trap immunoprecipitations of GFP alone, PAWS1–GFP and GFP–PAWS1 were resolved by SDS-PAGE, and sections covering the entire lane for each sample were excised and digested with trypsin (Fig. 4A). The resulting peptides were subjected to liquid chromatography tandem MS (LC-MS/MS) for identification. In addition to the SMAD isoforms, one of the most robust protein interactors identified for both GFP–PAWS1 and PAWS1–GFP but not GFP alone was CD2AP (Fig. 4A; Fig. S4A,C,D). CD2AP plays a role in controlling actin cytoskeletal dynamics and cell migration (Srivatsan et al., 2013; Tang and Brieher, 2013). We went on to verify the interaction between PAWS1 and CD2AP. Upon co-expression in HEK293 cells, Myc–CD2AP is detected in FLAG–PAWS1 immunoprecipitations but not in control FLAG immunoprecipitations (Fig. 4B). Endogenous PAWS1 was detected in GFP–CD2AP immunoprecipitations but not in control GFP immunoprecipitations from U2OS cells transiently transfected with either GFP–CD2AP or GFP (Fig. 4C). In order to verify endogenous interaction between PAWS1 and CD2AP, and without access to antibodies that can effectively immunoprecipitate PAWS1 and CD2AP, we generated homozygous PAWS1–GFP knockin U2OS cells by using CRISPR/Cas9 (Fig. 4D). We used an anti-GFP antibody to immunoprecipitate PAWS1–GFP from PAWS1–GFP-knockin U2OS cells and subjected the resulting material to MS (Fig. S4B,E). We detected CD2AP in these immunoprecipitations but not in those derived from PAWS1^−/−^ U2OS cells. Endogenous CD2AP and PAWS1 were also detected by western blotting in anti-GFP immunoprecipitations from PAWS1–GFP-knockin, but not WT U2OS cells transfected with GFP control (Fig. 4D). Taken together, these observations demonstrate an interaction between PAWS1 and CD2AP. To map the PAWS1 interaction domain, we co-expressed Myc-tagged PAWS1 fragments with full-length GFP–CD2AP in PAWS1^−/−^ cells and performed co-immunoprecipitation experiments (Fig. 4E). GFP–CD2AP co-precipitated PAWS1 only when PAWS1 contained residues 151–291, which are located within the DUF1669 domain (Fig. 4E). Consistent with these observations, when ∼100-amino-acid fragments of FLAG-tagged PAWS1 spanning the entire protein were co-expressed with full-length Myc-tagged CD2AP in PAWS1^−/−^ cells, only FLAG–PAWS1(204-294) and full-length FLAG–PAWS1 were able to co-immunoprecipitate Myc–CD2AP (Fig. S4D).

### PAWS1 colocalizes with CD2AP in cells

**Fig. 5.**
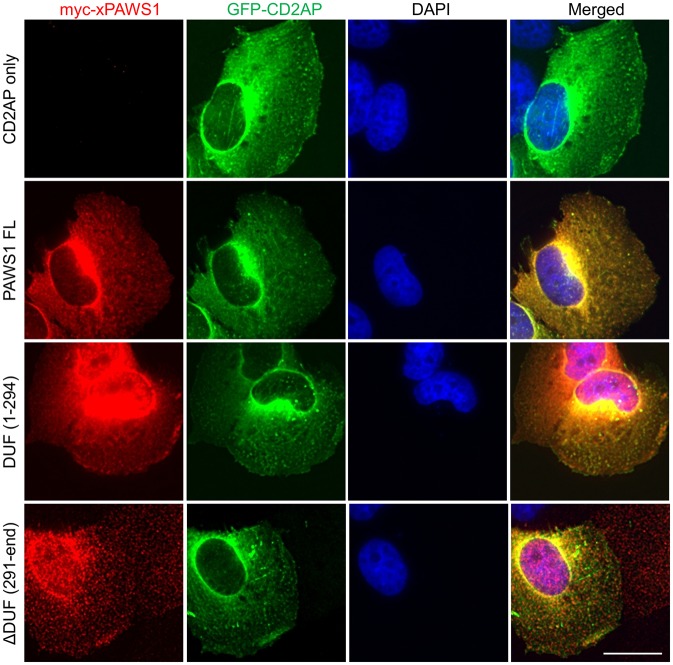
**PAWS1 and CD2AP colocalize in U2OS cells.** PAWS1^−/−^ U2OS cells were transfected with GFP–CD2AP alone, or co-transfected with the indicated fragments of Myc-tagged Xenopus PAWS1 (xPAWS1). Cells were fixed in PFA and subjected to immunofluorescence staining with anti-Myc-tag antibody, followed by Alexa Fluor-conjugated (red) secondary antibody. Fluorescence images for Myc–PAWS1 (red) and GFP–CD2AP (green) were captured using a DeltaVision system. *Z*-series were collected at 0.2 μm intervals, and deconvolved using SoftWoRx. *Z*-projections and image analysis were performed with OMERO. Scale bar: 20 μm.

To further confirm the interaction between CD2AP and PAWS1 in cells, we assessed the subcellular localization of GFP–CD2AP and Myc–PAWS1 fragments co-expressed in PAWS1^−/−^ U2OS cells (Fig. 5) by immunostaining and fluorescence microscopy on fixed cells. In the absence of PAWS1, GFP–CD2AP was localized predominantly in the cytoplasm. When co-expressed, a substantial overlapping cytoplasmic staining was observed for both full-length PAWS1 and GFP–CD2AP (Fig. 5). The DUF1669 domain (1–294) of PAWS1, which binds CD2AP (Fig. 4E), showed both nuclear and cytoplasmic staining but the overlapping staining with CD2AP was only observed in the cytoplasm (Fig. 5B), suggesting CD2AP colocalizes with PAWS1 only in the cytoplasm. When GFP–CD2AP was co-expressed with the interaction-deficient PAWS1(291-end) fragment (Fig. 4E), very little overlapping staining was observed (Fig. 5). A very distinct pan-cellular punctate staining for PAWS1(291-end) fragment was observed (Fig. 5). Taken together with the immunoprecipitation experiments, these data suggest robust interactions between CD2AP and PAWS1.

**Fig. 6.**
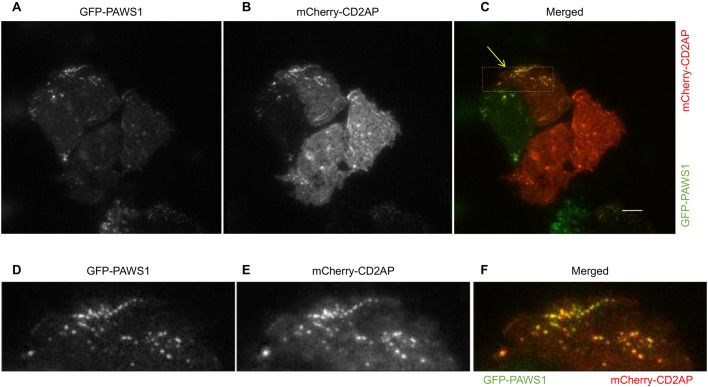
**CD2AP and PAWS1 colocalize in dynamic punctate structures proximal to the plasma membrane in WT U2OS cells.** TIRF microscopy in U2OS cells transfected with mCherry–CD2AP and GFP–PAWS1. (A) GFP–PAWS1, (B) mCherry-CD2AP and (C) merged mCherry–CD2AP and GFP–PAWS1 showing colocalization around the plasma membrane. Scale bar: 10 µm. (D–F) Magnifications of the boxed region indicated by an arrow in C.

**Fig. 7.**
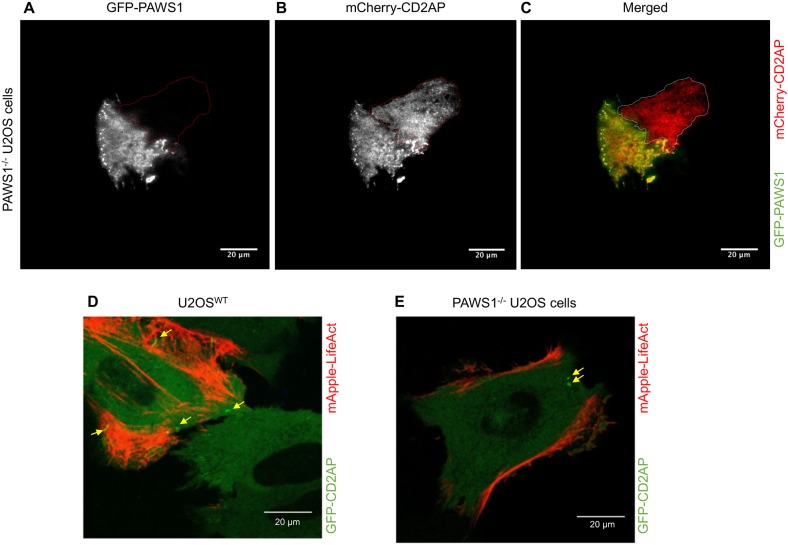
**PAWS1 appears to regulate localization of CD2AP with dynamic actin.** (A–C) PAWS1^−/−^ U2OS cells transfected with mCherry–CD2AP (red) and GFP–PAWS1 (green) for 24 h were followed by TIRF live-cell imaging for 25 min to assess the localization and dynamics of CD2AP and PAWS1 around the membranes. Note that cross-channel bleed-through is minimal, indicated by lack of cross channel GFP excitation/emission in B with only the mCherry-positive cell outlined. Representative images are shown. Scale bars: 20 µm. (D) WT U2OS and (E) PAWS1^−/−^U2OS cells were transfected with GFP–CD2AP (green) and mApple–LifeAct (red) for 24 h and followed by wide-field fluorescence microscopy live cell imaging at 40× magnification for 30 min. Representative images are shown. GFP–CD2AP puncta are indicated by yellow arrows. Scale bars: 20 µm.

Next, in order to investigate the dynamics of PAWS1–CD2AP interaction in cells, we used live-cell TIRF microscopy on WT U2OS cells transfected with GFP–PAWS1 and mCherry–CD2AP. In addition to cytoplasmic colocalization, under these conditions we observed that the two proteins colocalize in dynamic punctate structures adjacent to ruffling membranes and lamellipodia [Fig. 6 (C,F show the merged images); Movies 6–8]. Over the course of live-cell imaging, some non-overlapping, predominantly cytoplasmic staining of both GFP–PAWS1 and mCherry–CD2AP was also observed (Fig. 6). The dynamic colocalization of PAWS1 and CD2AP in distinct structures suggests there might be regulated interaction between these proteins. Interestingly, when the colocalization of transiently transfected GFP–PAWS1 and mCherry–CD2AP was explored by performing TIRF microscopy in PAWS1^−/−^ U2OS cells, similar overlapping punctate structures adjacent to ruffling membranes were observed (Fig. 7A–C), but in an adjacent PAWS1^−/−^ cell in which GFP–PAWS1 was absent, no punctate structures were visible for mCherry–CD2AP (Fig. 7A–C). These observations suggest that PAWS1 may be required for localization of CD2AP at the dynamic punctate structures.

To understand the impact of PAWS1 on CD2AP localization in the context of actin cytoskeletal dynamics, we analysed WT and PAWS1^−/−^ U2OS cells transfected with GFP–CD2AP and mApple–LifeAct by performing wide-field fluorescence microscopy (Fig. 7D,E). In WT U2OS cells, GFP–CD2AP puncta were visualized close to the active ruffling lamellipodia and actin-rich components of the plasma membranes (Fig. 7D). In contrast, in PAWS1^−/−^ cells GFP–CD2AP was distributed diffusely in the cytoplasm and any visible puncta did not extend into lamellipodia (Fig. 7E).

### CD2AP deficiency phenocopies the actin and migratory defects caused by PAWS1 deficiency

**Fig. 8.**
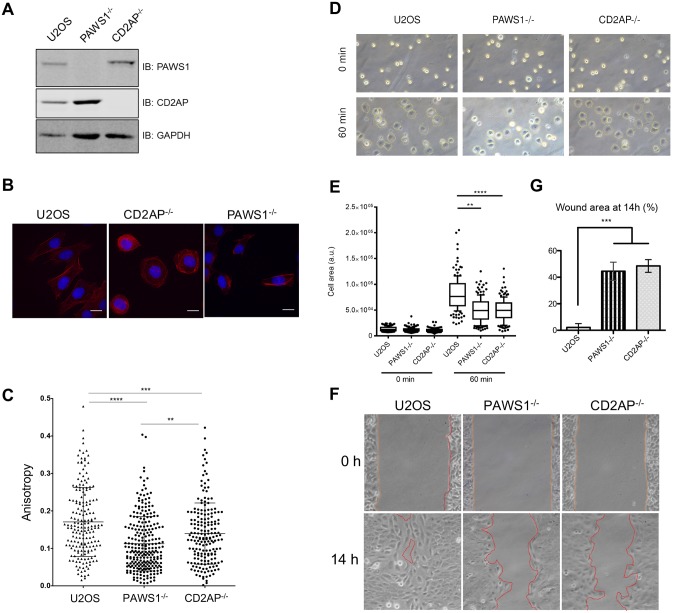
**PAWS1- and CD2AP-deficient U2OS cells exhibit reduced lamellar actin and impared migration.** (A) Loss of CD2AP protein in CD2AP^−/−^ cells was confirmed by immunoblotting (IB). (B) U2OS WT, CD2AP^−/−^ or PAWS1^−/−^ cells were fixed and stained with fluorescently labelled phalloidin after 24 h adhesion to glass coverslips under normal growth and serum-fed medium conditions. Images were acquired, then analysed by using ImageJ and the plug-in Fibriltool to assess organization of actin fibres and attain a value for anisotropy. Cells with multiple or overlapping nuclei were excluded. Scale bars: 20 µm. (C) Values are expressed as the anisotropy of actin fibres. Cells measured from three independent experiments in 15–20 coverslips per experiment and genotype, and the mean±s.d. is also shown. **P<0.01, ***P<0.001, ****P<0.0001 (one-way ANOVA with Tukey's multiple comparisons test). (D) Serum-starved cells were seeded onto fibronectin-coated slides and images were taken at 0 and 60 min. Representative images, with cell boundaries marked, are shown. (E) Cell areas were measured in ImageJ across three independent experiments as in D (*n*>150 cells/condition). Boxplots show the median, upper and lower quartiles. Whiskers indicate the 10th and 90th percentiles. **P<0.01, ****P<0.0001 (one-way ANOVA with Dunnett's test). (F) Representative images showing the wound gap at 0 and 14 h following removal of the insert separating wells of confluent cells. (G) Wound area from experiments as in F was measured in ImageJ and shown as a percentage of the area at 0 h. Values are the mean±s.d. of three independent experiments. ***P<0.001 (one-way ANOVA with Dunnett's test).

To investigate the role of CD2AP in actin cytoskeleton and cell migration relative to PAWS1, we generated CD2AP-knockout U2OS cells (CD2AP^−/−^) by CRISPR/Cas9, targeting the exon 3 of the CD2AP gene. The loss of CD2AP protein in the isolated clone of U2OS cells was verified by western blotting (Fig. 8A), and the genomic alterations at the target loci were verified by genomic sequencing. We first analysed actin distribution in WT, PAWS1^−/−^ and CD2AP^−/−^ U2OS cells by performing phalloidin staining. Visually, the distribution of actin in PAWS1^−/−^ and CD2AP^−/−^ cells was similar but different from that in the WT U2OS cells (Fig. 8B). We also measured the actin-positive areas within the cells for anisotropy. The scores ranged between 0 and 1, with 0 defined as disordered (isotropic) actin structures and 1 defined as completely ordered (anisotropic) actin structures (Boudaoud et al., 2014). The results showed that actin was significantly more ordered in WT U2OS cells than in either PAWS1^−/−^ or CD2AP^−/−^ U2OS cells (Fig. 8C). In order to assess whether adhesion and spreading of PAWS1^−/−^ and CD2AP^−/−^ cells relative to the WT U2OS cells were affected, we seeded these cells on fibronectin-coated plates and measured the cell areas from images taken at 0 and 60 min after seeding (Fig. 8D,E). Compared to WT U2OS cells, the measured cell areas of both PAWS1^−/−^ and CD2AP^−/−^ cells at 60 min were significantly smaller (Fig. 8D,E), suggesting that loss of either PAWS1 or CD2AP causes reduced spreading of cells upon attachment. To assess the role of CD2AP in cell migration, we performed a lateral wound-healing assay by using WT, PAWS1^−/−^ and CD2AP^−/−^ U2OS cells cultured to confluency in adjacent chambers of a culture well divided by a small fixed-sized spacer, as in the experiments above (Fig. 1D). The migration of cells into the gap was monitored at 0 and 14 h (Fig. 8F,G). As shown above, significantly fewer PAWS1^−/−^ cells migrated into the gap than WT cells (Fig. 8F,G). Interestingly, significantly fewer CD2AP^−/−^ cells had also migrated into the gap at 14 h than WT cells (Fig. 8F,G). Collectively, these results suggest that CD2AP and PAWS1 both play key, and possibly synergistic, roles in actin distribution, cell spreading and cell migration in U2OS cells.

## DISCUSSION

Our previous work has shown that PAWS1 interacts with SMAD1, that it is a substrate of type I BMP receptor kinases, and that it is involved in Smad4-independent BMP signalling. We also demonstrated that PAWS1 regulates the expression of several non-BMP target genes, suggesting that it has roles beyond the BMP pathway (Vogt et al., 2014). By knocking out PAWS1 from U2OS osteosarcoma cells, we show here that PAWS1 plays a role in actin organization, morphology, spreading and migration in U2OS cells, and that it is likely to exert these effects through its interaction with CD2AP. In particular, the interaction of PAWS1 with CD2AP at the cell periphery appears to control actin dynamics to initiate lamellipodia formation and cellular migration. Indeed, U2OS cells with CD2AP deficiency also exhibit actin cytoskeletal and cell migratory defects reminiscent of those in PAWS1-knockout U2OS cells. Future work will explore the mechanisms through which the association of PAWS1 and CD2AP control the actin cytoskeleton, and the ability of PAWS1 to influence both BMP signalling and the actin cytoskeleton, to ask whether the two functions are linked and to ask whether any other activities can be attributed to PAWS1.

Understanding the biochemical and molecular bases for the regulation of cytoskeletal architecture has important implications for key biological processes, including embryonic development, angiogenesis, fibrosis and the epithelial–mesenchymal and mesenchymal–epithelial transitions, among many others. Cells require dynamic and finely tuned molecular machinery to initiate, prolong and execute locomotion and migration processes in response to external stimuli. External stimuli, including growth factor signals, mechanical stress and cell–cell contacts, can modulate chemotactic or haptotactic responses, directing cellular motility and invasive potential. Changes in cell shape impact on the locomotor and motile properties of a given cell. Two major processes involved in locomotion are derived from subcellular communication between the leading edge and the trailing edge of the cell. Lamellipodial protrusions at the leading edge define the directionality and intensity of locomotion and are the sites where focal adhesions form to anchor the protruded membrane to the extracellular matrix. Actin stress fibres that anchor to focal adhesions provide connections between the leading and trailing edges of the cells and provide the tracks that myosin motors use for generating contractile forces. Focal adhesions that are engaged with integrins become platforms for intracellular signalling cascades to propel changes in actin dynamics. Our findings suggest key roles for both PAWS1 in focal adhesion dynamics and actin cytoskeletal organization processes that ultimately control cellular migration.

Precisely how PAWS1 acts to impart its effects on cytoskeletal organization and cellular migration remains to be defined. However, our data implies that its association and dynamic colocalization with the multifunctional scaffold protein CD2AP could play an important part. CD2AP has been reported to associate with cortactin and capping proteins, directing them to the barbed ends of polymerizing F-actin at the cell periphery to enable actin assembly for lamellipodia formation (Bruck et al., 2006; Srivatsan et al., 2013; Zhao et al., 2013). Clearly, in the absence of PAWS1, it appears that CD2AP cannot accumulate at the cell periphery, and this could explain the actin assembly and organization defects, especially those affecting lamellipodia. It is therefore likely that the dynamic interaction of PAWS1 and CD2AP at the membrane ruffles close to the lamella plays a key role in the assembly of productive actin networks. Large pools of non-overlapping PAWS1 and CD2AP exist within the cytoplasm, suggesting that their interactions at the cell periphery could be regulated, perhaps through specific signalling cues. Understanding these and the molecular determinants of PAWS1–CD2AP interactions is essential for establishing whether the association between PAWS1 and CD2AP is essential and sufficient for coordinating actin assembly and cytoskeletal organization. Currently, we do not fully understand the precise biochemical roles of PAWS1. It is possible that PAWS1 acts as a scaffold protein to recruit key factors to the PAWS1–CD2AP complex to initiate actin reorganization at the membrane ruffles.

Dynamic cytoskeletal reorganization and regulated cell migration in response to specific signalling cues are fundamental cellular processes during embryonic development and in adult tissue homeostasis. A common feature in cancer cells is the adoption of aggressive migratory behaviour through dysregulation of cytoskeletal components such as actin dynamics and organization that promotes adaptive advantages of malignant tumour cell migration and invasion. Our findings identify PAWS1 as a novel regulator of the dynamic actin cytoskeletal network and cell migration. A better molecular understanding of how PAWS1 impacts on cell migration could uncover therapeutic opportunities to target metastatic cancers that exploit dysregulated migratory processes.

## MATERIALS AND METHODS

### Antibodies

An anti-FAM83G antibody was produced by the Dundee Division of Signal Transduction and Therapy (DSTT) as a sheep polyclonal antibody against the C-terminus of FAM83G (S876C); sheep anti-GFP was also produced by the DSTT (S268B). Other antibodies used in these studies were: Myc tag (cat. no. CST2276; Cell Signaling Technology); FLAG-M2-HRP (cat. no. A8592; Sigma); MYC-HRP (cat. no. 11814150001; Roche); CD2AP (gift from Andrey Shaw, Research Biology, Genentech, South San Francisco, CA), CD2AP (clone 2A2.1, cat. no . MABT419; Millipore), anti-FAM83G (cat. no. HPA023940; Sigma), and actin (cat. no. ab8227; Abcam). Secondary rabbit, mouse and sheep antibodies conjugated to horseradish peroxidase (HRP) were used at 1:10,000 (Santa Cruz Biotechnology). Fluorophore-conjugated phalloidin (Alexa Fluor 488 and Atto 562) and Alexa-Fluor-594-conjugated anti-mouse-IgG (ThermoFisher) were used for fluorescence microscopy.

### Cell culture

Cells (U2OS, 293T and HEK293) (originally sourced from ATCC; modifications indicated where appropriate) were cultured in Dulbecco's modified Eagle's medium (DMEM; Gibco) with 10% FBS (Hyclone), 1% penicillin-streptomycin (Lonza) and 2 mM L-glutamine (Lonza). 293T cultures were supplemented with sodium pyruvate after onset of retrovirus production. All cells in culture were routinely tested for mycoplasma contamination and verified as mycoplasma negative.

### Vectors

Plasmids were designed and cloned in the DSTT, and site-directed mutagenesis was used to generate mutant forms of PAWS1. Other plasmids used were pBABE-PAWS1-puro, pBABE, pCDNA5-frt-TO-nGFP-PAWS1, pCDNA5-frt-TO-PAWS1-cGFP, pCDNA5-frt-TO-GFP, pCMV-GFP-CD2AP, mCherry-xCD2AP, GFP-CD2AP, mApple-LifeAct (Life Technologies), Emerald-LifeAct (Life Technologies), RFP–zyxin (provided by Yu-Li Wang, Department of Biomedical Engineering, Carnegie Mellon University, Pittsburgh, PA), pCS2-xPAWS1, pCS2-xCD2AP, pCDNA-PAWS1-FLAG (all made by co-authors and available on request. Xenopus (x) CD2AP, xPAWS1 (KSD) and all other constructs were made in the Division of Signal Transduction Therapy (DSTT), University of Dundee, UK). All DNA constructs were verified by DNA sequencing, performed by the DNA Sequencing & Services (MRCPPU, College of Life Sciences, University of Dundee, Scotland, http://www.dnaseq.co.uk) using Applied Biosystems Big-Dye Ver 3.1 chemistry on an Applied Biosystems model 3730 automated capillary DNA sequencer. All constructs are available to request from the MRC-PPU reagents webpage (http://mrcppureagents.dundee.ac.uk).

### FAM83G and CD2AP knockout via CRISPR/Cas9 genome editing

CRISPR/Cas9-mediated deletion of FAM83G/PAWS1 in osteosarcoma cells (U2OS) was performed by using Cas9 and a single guide (g)RNA targeting approach to delete exon 2 of the RefSeq gene for FAM83G (NM_001039999.2). Vectors containing the Cas9 and FAM83G-targeting gRNA (5′-GGACCGCTCCATCCCGCAGCTGG-3′) were transfected into 10^6^ U2OS cells followed by selection with 2 μg/ml puromycin and single cell sorting to isolate clone candidates with gene deletion. Sequencing of the gRNA targeting region indicated a 5-bp deletion causing a frameshift in the *FAM83G* gene. To knockout CD2AP (NM_012120.2), the Cas9 D10A ‘nickase’ mutant and paired gRNAs (5′-GTACAACGAATAAGCACCTA-3′ and 5′-GCCCATGCCTTTCCCGTTTGA-3′) approach (Ran et al., 2013) was used to target exon 3 of CD2AP. The resulting CD2AP-knockout clone yielded a 20-bp deletion, a 16-bp deletion and a 19-bp insertion. All mutations caused frameshifts leading to premature stop codons.

### Retroviral FAM83G/PAWS1 expression

Retroviral constructs of pBABE-puromycin, pBABE-PAWS1 or pBABE-GFP (5 µg each) were co-transfected with pCMV-gag/pol (4.5 µg) and pCMV-VSVG (0.5 µg) by using polyethylenimine (PEI, 1 mg/ml; 25 µl) in 1 ml OPTIMEM low-serum medium into a 10-cm dish of HEK293T cells. After 40 h of culture, supernatant medium was filtered (0.45 µm) and applied to recipient cells and supplemented with 8 µg/ml polybrene (Sigma #H9268, Hexadimethrine bromide**)**. Recipient U2OS cells were plated at 40–50% confluence and then infected with the indicated virus for 24 h. Following virus infection, U2OS cells were treated with puromycin at 2 µg/ml to select for vector integration by the virus.

### Two-dimensional lateral cell migration

U2OS cells were plated into ibidi insert chambers (Cat# 80209) for 18 h before two-dimensional migration assays were performed. Equal numbers (40,000–60,000) of cells were plated on both sides of the chamber and the silicone insert was removed to allow lateral migration. Cells were incubated in a 5% CO_2_-regulated and 37°C temperature-controlled chamber. Images were collected for 18–24 h with a Nikon Eclipse Ti microscope. Images of the wound gap were collected every 5 min by a Photometrics Cascade II CCD camera with Nikon NIS elements software. Wound closure was measured with ImageJ and reported as a percentage of closure relative to the starting wound size.

### Cell spreading and chemotaxis assays

For cell spreading assay, WT, PAWS1^−/−^ or CD2AP^−/−^ U2OS cells were serum-starved for 16 h, trypsinized and introduced into a µ-Slide chamber (Ibidi, Cat#80601) at a density of 3×10^5^ cells/ml. Slides were pre-coated with fibronectin (Sigma, F4759) according to manufacturer's recommendation. Images from multiple fields of view in duplicate chambers for each cell line were taken at 0 and 60 min using a digital camera attached to a phase-contrast microscope. Cell boundaries were marked, and areas were measured with ImageJ. Dead or dying cells and closely packed cells were excluded from the analysis. Analysis was performed on images from three independent experiments. For chemotaxis assays, cells were introduced into one end of a chamber at a density of 3×10^6^ cells/ml, while the opposite end was loaded with medium containing 10% FBS (Pepperell and Watt, 2013). Images of migrating cells were collected every 5 min on with a Nikon Eclipse Ti microscope and Photometrics II CCD camera. For quantification purposes, cells were scored based on phenotypes defined as non-adhesive, adhesive with some attachment, adhesive without lamella projections and adhesive with lamella projections.

### F-actin staining

U2OS cells were seeded onto microscope slides at low density and allowed to grow to 20–30% confluence. Cells were then fixed with 4% paraformaldehyde (PFA) for 30 min, and washed twice in DMEM/HEPES pH 7.4 followed by 10 min incubation in DMEM/HEPES. Cells were washed once in PBS, then in 0.2% Triton X-100 in PBS for 3–5 min. Cells were washed in 1% bovine serum albumin (BSA) in PBS followed by staining with Phalloidin (Alexa-Fluor-488 or Atto-562) performed at 1:500 dilution in the dark for 1 h at room temperature. Following incubation, slides were washed 3× in BSA/PBS solution. Coverslips were mounted in Prolong gold with DAPI for nuclear staining. Coverslips were allowed to dry briefly then sealed and imaged by widefield deconvolution microscopy. To assess cell actin organization in phalloidin-stained WT, PAWS1^−/−^ and CD2AP^−/−^ U2OS cells, the actin area was bounded in ImageJ then submitted to the plugin Fibriltool to determine anisotropy in response to genotype (Boudaoud et al., 2014). Overlapping cells or those undergoing division were omitted from analysis.

### Immunofluorescence

Cells were fixed with 4% PFA in PBS for 10 min, and permeabilized with 0.5% Triton X-100 in PBS for 5 min. Coverslips were incubated in blocking buffer (3% BSA, 0.1% Triton X-100 in PBS) for 30 min, followed by primary antibodies diluted in blocking buffer for 1 h. Cells were washed with 0.1% Triton X-100 in PBS, and incubated with the appropriate Alexa Fluor-conjugated secondary antibodies (ThermoFisher Scientific). Images were captured using a DeltaVision system (Applied Precision). *Z*-series were collected at 0.2 μm intervals, and deconvolved using SoftWoRx (Applied Precision). *Z*-projections and image analysis were performed with OMERO (www.openmicroscopy.org).

### Transfection of fluorescent proteins

Cells were transfected with 2–5 µg of GFP–PAWS1, mCherry–CD2AP, RFP–Zyxin (a gift from Yu-li Wang, Department of Biomedical Engineering, Carnegie Mellon University, Pittsburgh, PA), mApple–LifeAct (Invitrogen) or Emerald LifeAct (Invitrogen) along with Fugene HD or PEI. Cells were cultured for 24–48 h and then imaged or processed as indicated.

### Live-cell imaging

U2OS cells were plated onto polystyrene CellView Culture (Greiner Bio-One) glass bottom dishes. Following transfection, images were captured by using a Zeiss LSM 700 confocal microscope in a regulated chamber with 5% CO_2_ at 37°C for 1–2 h as indicated. Images were taken of each fluorophore in sequence at 5 min increments by using Zen software.

### TIRF live-cell imaging

TIRF microscopy was used to detect interactions between PAWS1 as well as CD2AP and the actin cytoskeleton at the plasma membrane. Cells were plated in World Precision Instrument imaging chambers and transiently transfected with fluorophore-tagged PAWS1, CD2AP, LifeAct actin trackers (mApple or mEmerald) and RFP–zyxin and imaged in CO_2_-independent medium (Leibovitz's L-15; Life Technologies). TIRF was performed on a Nikon Ti-U microscope with an environmental control chamber (Okolab, Pozzuoli, Italy), a PAU/TIRF slider, 63× and 100×1.49 NA objectives, a PerfectFocus system, and a custom-built four-colour (405 nm, 488 nm, 561 nm, 647 nm) diode laser (Coherent Inc., Santa Clara, CA) system with a Gooch and Housego (Ilminster, UK) AOTF shutter (Solamere Technology, Salt Lake City, UT), an emission filter wheel (Nikon) with appropriate filters for eliminating crosstalk between channels (Chroma Technology Corp, Bellow Falls, VT) and a Photometrics Evolve Delta camera (Tucson, AZ). Images were all captured with µ-Manager (Open Imaging Inc., San Francisco, CA). Quantification of focal adhesion distribution in WT and PAWS1^−/−^ U2OS cells was determined by assigning an arbitrary internal cellular boundary close to the cellular periphery and measuring the number of RFP–Zyxin puncta either inside (internal) or outside (peripheral) the boundary with ImageJ. The data were plotted as a percentage of total focal adhesion puncta in each compartment.

### Micropattern analysis and wide-field fluorescence microscopy

Micropattern chips were from CYTOO (Grenoble, France) in multi-shape patterns including a crossbow, H-pattern and Y-pattern. The CYTOO 22-chip was coated with 20 µg/ml fibronectin in PBS for 2 h at room temperature according to the manufacturer's recommendation. The chip was washed three times in PBS and then air-dried overnight at 4°C. U2OS cells were split and plated onto the chip then washed after 1 h of attachment to minimize cytophobic surface binding. Cells were then fixed, stained with phalloidin conjugated to Alexa Fluor 562 or 594 and DAPI, and *z*-stacks were collected on a widefield deconvolution (GE Healthcare Life Sciences) or LSM 700 confocal microscope. Image analysis was performed with ImageJ on images acquired with equivalent exposure times for each experiment. F-actin accumulation in 10–15 WT or PAWS1^−/−^ U2OS cells was quantified in the lamellipodia and the radial arms in the ‘trailing ventral arms’ of the cells fixed on crossbow micropatterns. Cells also attached and formed stress fibres in the H pattern, and images were collected and analysed in a similar manner.

### Cell lysis, affinity purification and western blotting

Cells were washed twice in ice-cold PBS then scraped on ice into lysis buffer [50 mM Tris-HCl pH 7.5, 0.27 M sucrose, 150 mM NaCl, 1 mM EGTA, 1 mM EDTA, 1 mM sodium orthovanadate, 1 mM sodium β-glycerophosphate, 50 mM sodium fluoride, 5 mM sodium pyrophosphate, 1% (v/v) Triton X-100 and 0.5% Nonidet P-40] supplemented with complete protease inhibitor cocktail tablet (Roche). Lysates were clarified by centrifugation at ∼17,000 ***g*** at 4°C. Protein concentration was estimated through a Bradford assay (ThermoFisher). Typically, 15–30 µg was used for SDS-PAGE and 250 µg–1 mg extract proteins was used for immunoprecipitation and interaction studies. For immunoprecipitation, extracts were loaded with 10 µl of GFP trap beads (ChromoTek) or anti-FLAG-M2 beads (Sigma) and incubated on a rotator for 4–16 h at 4°C. Beads were washed in lysis buffer including 0.25 M NaCl once, followed by lysis buffer. Purified proteins were eluted in 1× sample buffer (50 mM Tris-HCl pH 6.8, 10% SDS, 50% glycerol, 0.1% Bromophenol Blue with 0.1% β-mercaptoethanol) and heated to 95°C for 5 min, and 25–50% of sample was fractionated in 4–20% or 10% SDS-PAGE gels as indicated. Gels were electroblotted onto Immobilon PVDF (Millipore) and blocked in 5% milk with TTBS (50 mM Tris-HCl pH 7.5, 0.2% Tween-20 and 150 mM NaCl) for 1 h at room temperature. Immunoblotting was performed with antibody at 1 µg/ml overnight in either 5% milk in TTBS or 5% BSA in TTBS at 4°C on a shaker. Blots were washed four times in TTBS and probed with secondary antibody to rabbit, sheep or mouse IgG conjugated to HRP (Santa Cruz Biotechnology) at 1:10,000 dilution in 5% milk in TTBS for 1 h at room temperature. Membranes were then washed four times with TTBS followed by enhanced chemiluminescent detection (ThermoFisher) and exposure to X-ray film or on a Gel Doc XR+ system using Image Lab software.

### MS analysis

GFP, GFP–PAWS1 or PAWS1–GFP constructs integrated stably into 293 TRex cells (Invitrogen) were expressed upon treatment with 20 ng/ml doxycyline for 16 h. Proteins were affinity purified with GFP-trap beads (ChromoTek) and subjected to MS analysis as previously described (Vogt et al., 2014). Briefly, purified proteins were separated by SDS-PAGE on 4–12% gradient gels then stained with colloidal Coomassie Blue overnight. The gel was washed in distilled water until background staining was minimal. Six gel pieces covering the entire lanes for each pulldown were excised, trypsin digested, and peptides prepared for HPLC gradient fractionation and elution into a Thermo Scientific Velos Orbitrap mass spectrometer. Ion assignments were conducted by in-silico Mascot scoring (www.matrixscience.com) and peptide protein assignments were reported in Scaffold 4.1 (www.proteomesoftware.com).

## Supplementary Material

Supplementary information
