# Supplementary material for: PAWS1 controls cytoskeletal dynamics and cell migration through association with the SH3 adaptor CD2AP
Source: J Cell Sci. 2018 Jan 1;131(1):jcs202390. doi: 10.1242/jcs.202390 (PMC5818054; doi:10.1242/jcs.202390)
Supplement: Supplementary information [file joces-131-202390-s1.pdf]

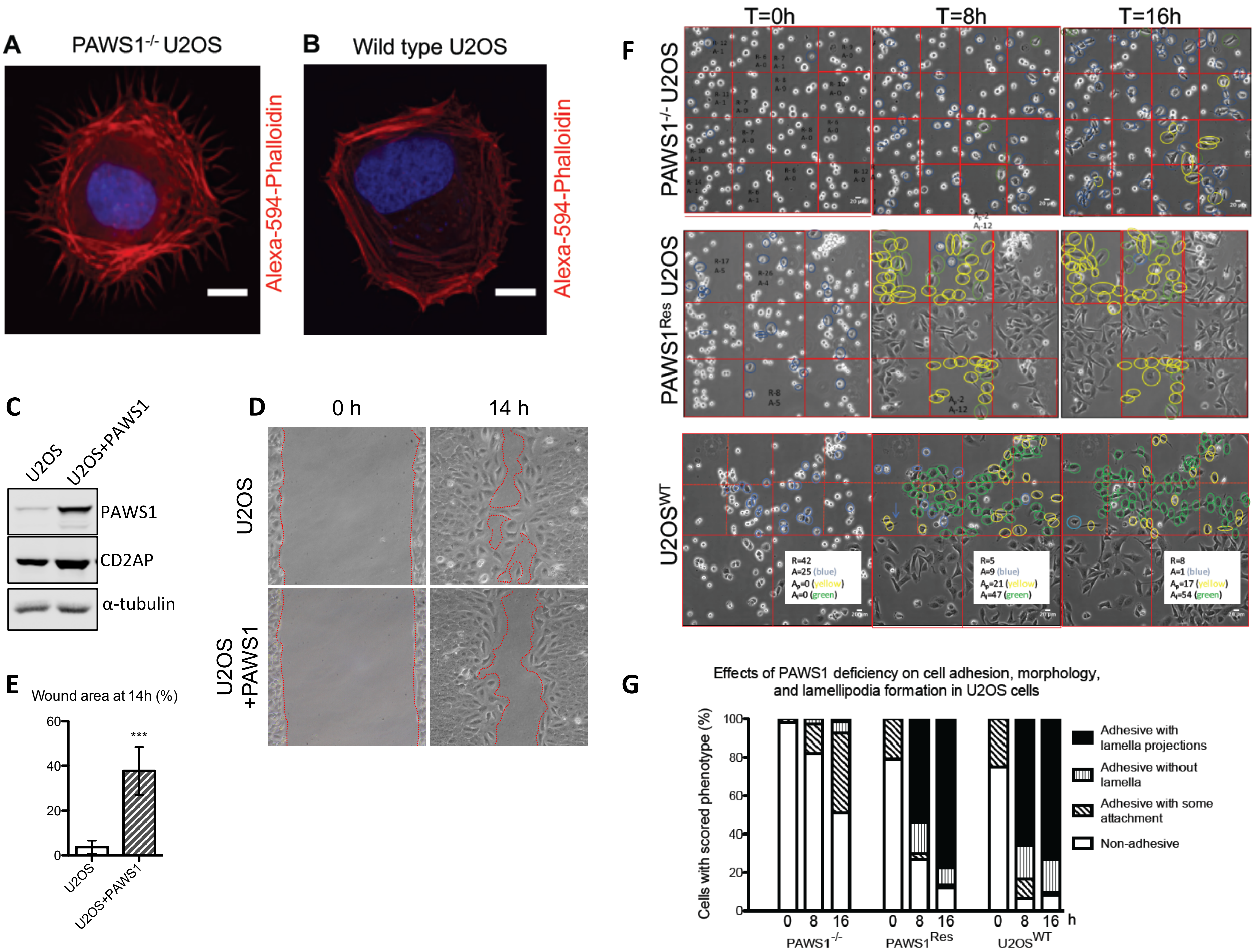

Journal of Cell Science • Supplementary information

Supplementary Figure 1. Analysis of actin fibres in wild type and PAWS1$^{-/-}$ U2OS cells and chemotaxis assay for wild type U2OS, PAWS1$^{-/-}$ U2OS and PAWS1^{Res} U2OS cells with FBS. A. PAWS1$^{-/-}$ U2OS cells and **B.** wild type U2OS were fixed and stained with Alexa-594-Phalloidin (red). Representative images are included. Scale bars indicate 10 μm. **C.** Western blot of extracts from U2OS cells overexpressing PAWS1 via infection with retroviral vectors. **D.** Representative images showing the wound gap at 0 and 14 h following removal of the insert separating wells of confluent cells. **E.** Wound area was measured in ImageJ and shown as a percentage of the area at 0 h. Values are the Mean±S.D. of 3 independent experiments. Student's t-test (***P<0.001). **F&G.** Quantification of cell adhesion and morphology. Freshly trypsinized wild type (^{WT}), PAWS1$^{-/-}$, PAWS1^{Res} U2OS cells were seeded into a μ-Slide chemotaxis chamber at one end at 3x10^{6} cells/ml while the opposite end was loaded with DMEM medium containing 10% FBS. For images acquired at 0 h, 8 h, and 16 h after seeding by a Photometrics II CCD camera with Nikon NIS elements software, arbitrary grids (representative images of PAWS1$^{-/-}$, PAWS1^{Res} and wild type are shown in **C**) were assigned and within each grid, all cells were scored based on phenotypes as follows: non-adhesive (R; rounded, non-attached), adhesive with some attachment (A; appearing rounded but beginning to attach), adhesive without lamella projections (A_p; attached cells without distinct lamella projections), and adhesive with lamella projections (A_L). The number of cells in each category were then counted for PAWS1$^{-/-}$, PAWS1^{Res} and wild type U2OS cells from at least three different grids for each time point and plotted as a percentage relative to all assigned phenotypes **(F)**.

Journal of Cell Science • Supplementary information

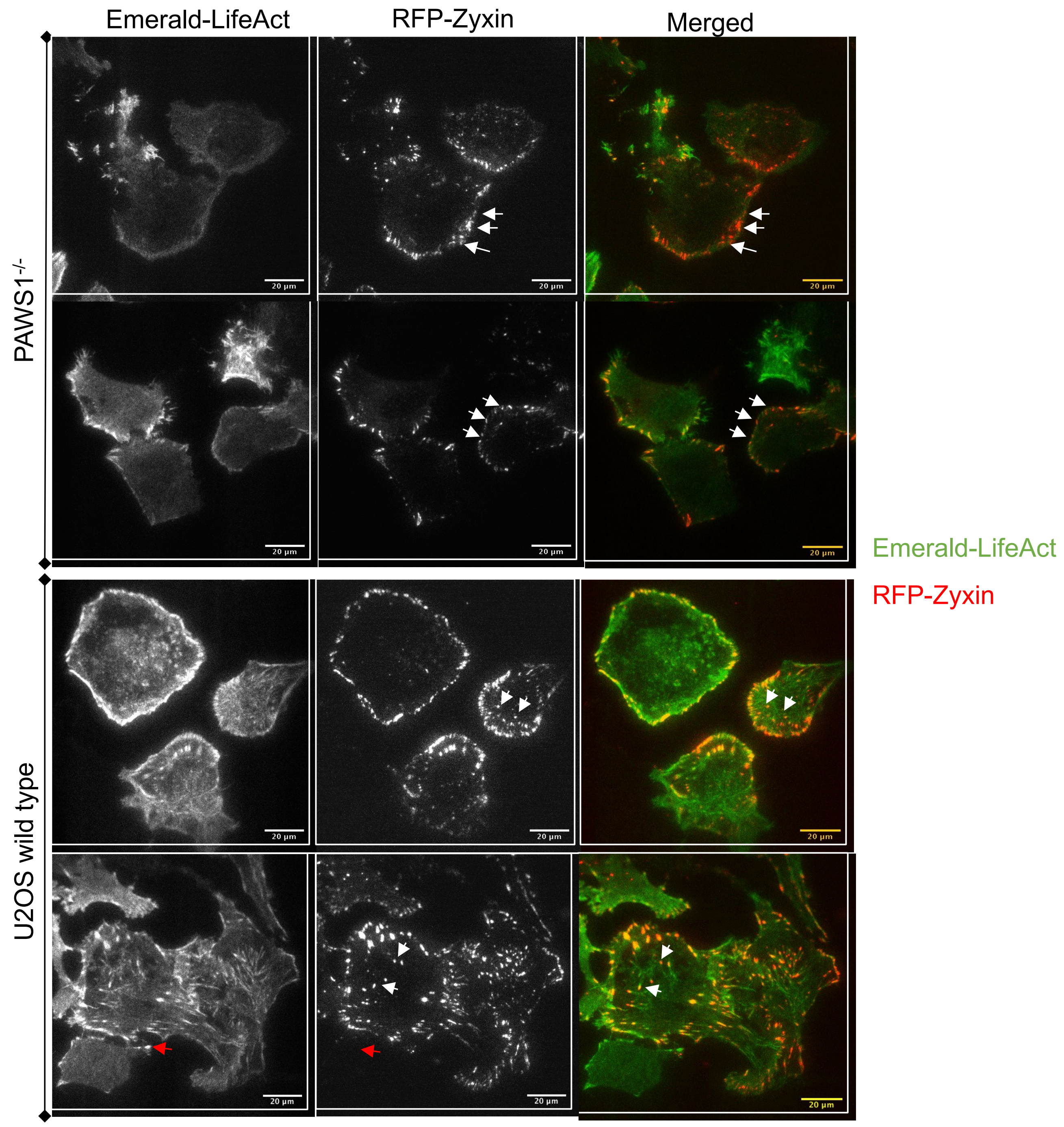

Supplementary Figure 2. Analysis of focal adhesion dynamics in PAWS1$^{-/-}$ and wild type U2OS cells using TIRF microscopy. PAWS1$^{-/-}$ (upper panel) and wild type (lower panel) U2OS cells were transfected with RFP-zyxin (red) and Emerald-LifeAct (green) to measure changes in cytoskeletal and focal adhesion dynamics. Arrows indicate focal adhesion points. Panels are duplicate representative images. TIRF microscopy was conducted for the analysis of membrane dynamics of the adhesion process.

Journal of Cell Science • Supplementary information

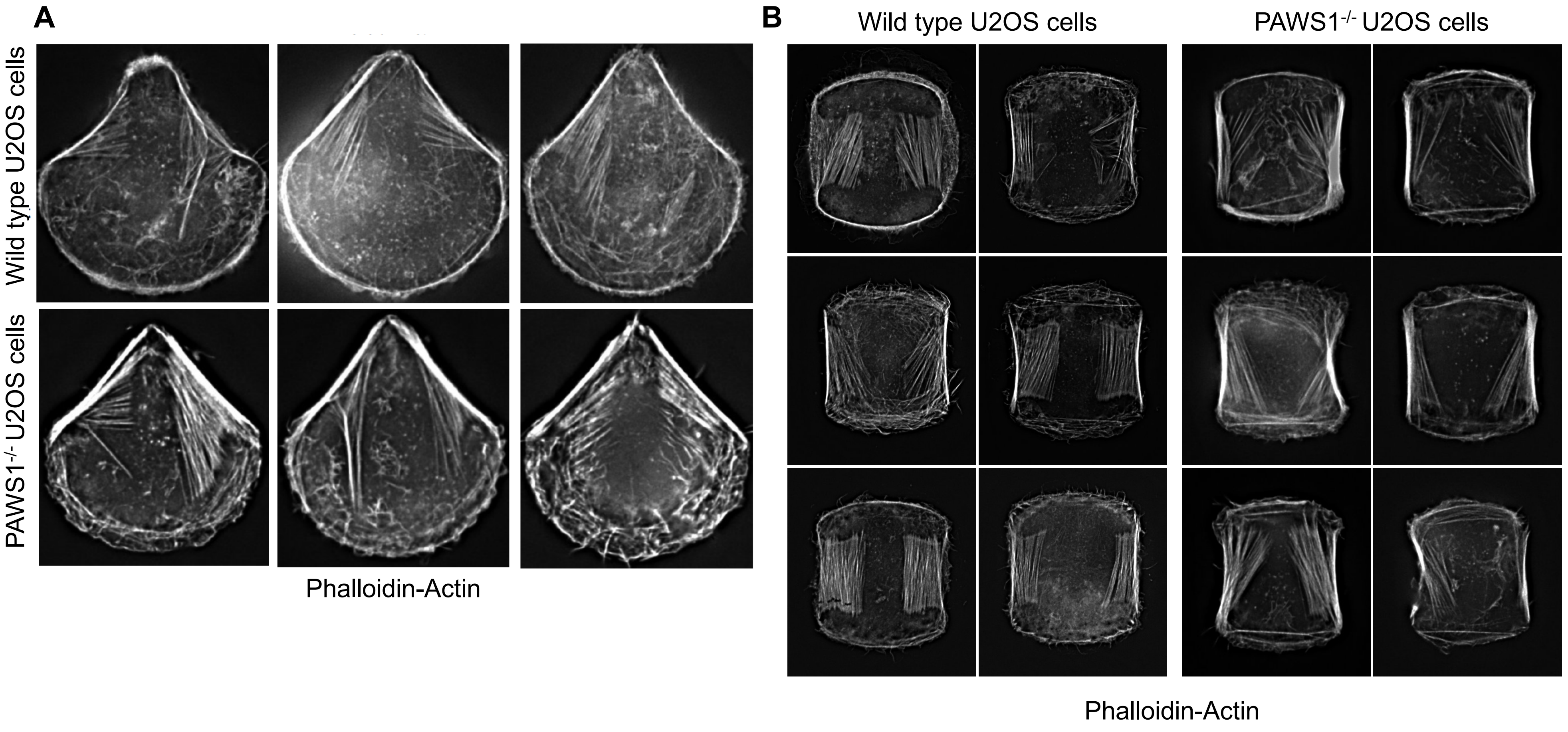

Supplementary Figure 3. Micropattern images of PAWS1 deficient and wild-type cells. **A&B.** Additional representative images of micropattern adhesion dynamics of phalloidin-stain in wild type and PAWS1$^{-/-}$ U2OS cells in the crossbow **(A)** and double crossbow **(B)** patterns. Monochrome images of each genotype are presented to indicate the shape and structure of actin fibres. Cells and images were processed as described in Legends to Figure 3A and C.

A

Protein ID	Total Spectral Counts in anti-GFP IPs (from cells transfected with)		
	GFP (TC140919)	GFP-PAWS1 (TC140919)	PAWS1-GFP (TC140123)
PAWS1	8	664	903
CD2AP	1	165	283

B

Protein ID	Total Spectral Counts in anti-GFP IPs	
	PAWS1$^{-/-}$ (PB150715)	PAWS1$^{GFP/GFP}$ (PB150715)
PAWS1	2	75
CD2AP	0	7

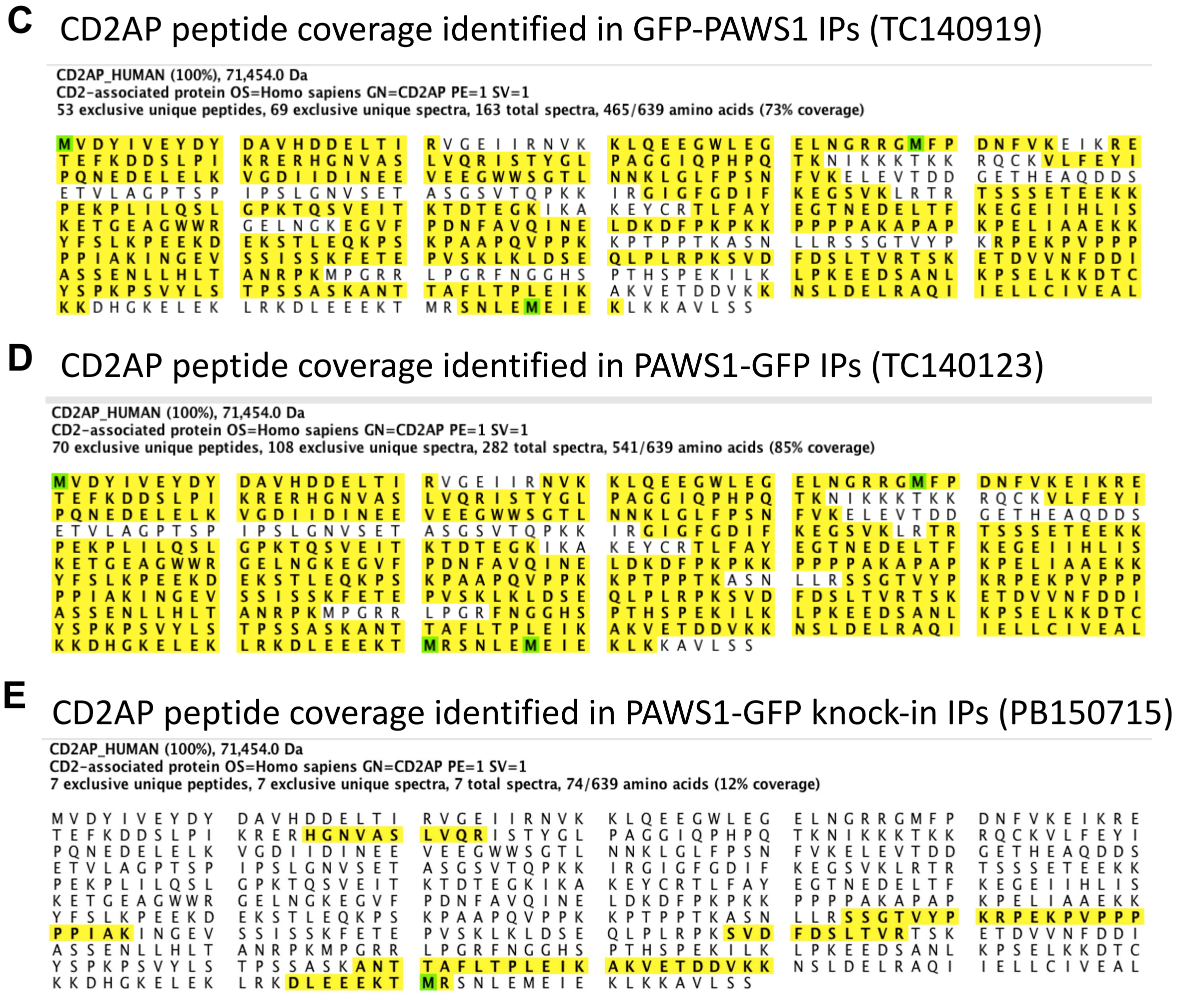

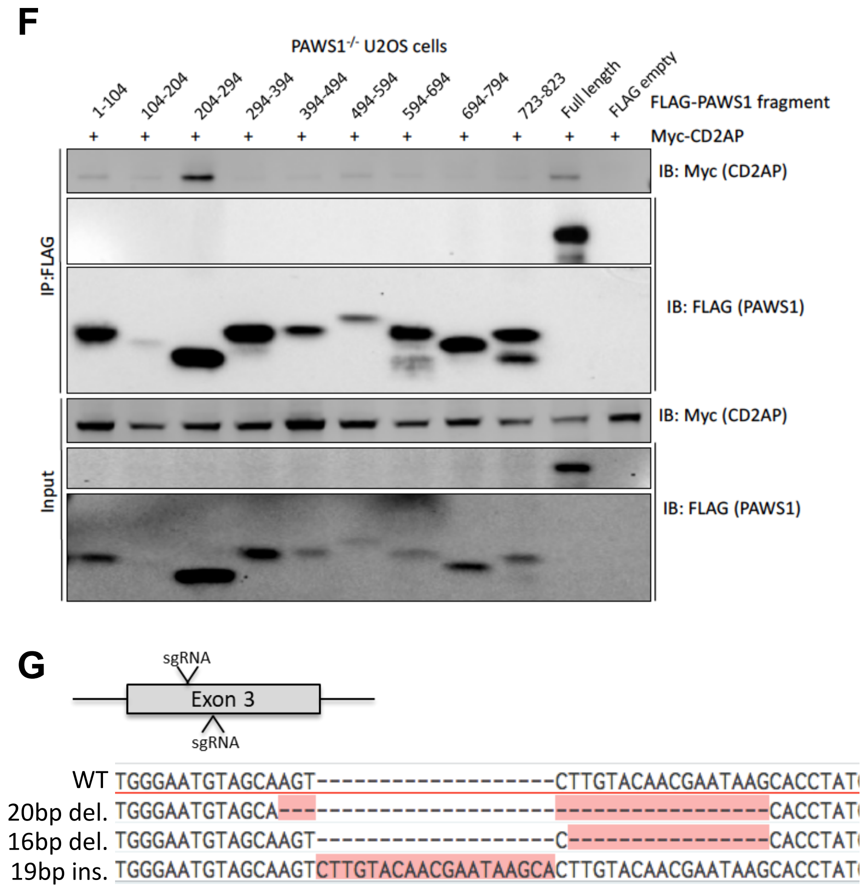

Supplementary Figure 4. Analysis of PAWS1-CD2AP interactions. A. Mass fingerprinting of protein interactors of GFP-PAWS1 and PAWS1-GFP IPs but not of GFP IPs deployed as control (Fig. 4A) identified PAWS1 and CD2AP. **B.** GFP IPs from PAWS1$^{-/-}$ and homozygous PAWS1-GFP knockin U2OS cells (PAWS1$^{GFP/GFP}$) were subjected to the same analysis. Both A and B show the total spectral counts obtained for PAWS1 (bait) and endogenous CD2AP (interactor) in each IP sample. Scaffold Q/Q+S V4.4.6 was used to analyse the Mascot search results derived from the LC-MS-MS data. **C-E.** The peptide coverage of CD2AP in GFP-PAWS1 (C), PAWS1-GFP (D), and PAWS1$^{GFP/GFP}$ (E) IPs are indicated. **F.** Mapping PAWS1-CD2AP interactions. The indicated fragments of Flag-tagged PAWS1 were co-expressed with Myc-tagged full-length CD2AP in PAWS1$^{-/-}$ U2OS cells for 48 hrs. Extracts or anti-Flag IPs were subjected to immunoblotting with anti-myc (CD2AP) and anti-Flag (PAWS1) antibodies as indicated. **F.** CRISPR mediated deletion of CD2AP at exon 3 was confirmed by sequencing of genomic DNA around the targeted region.

Journal of Cell Science • Supplementary information

Movies

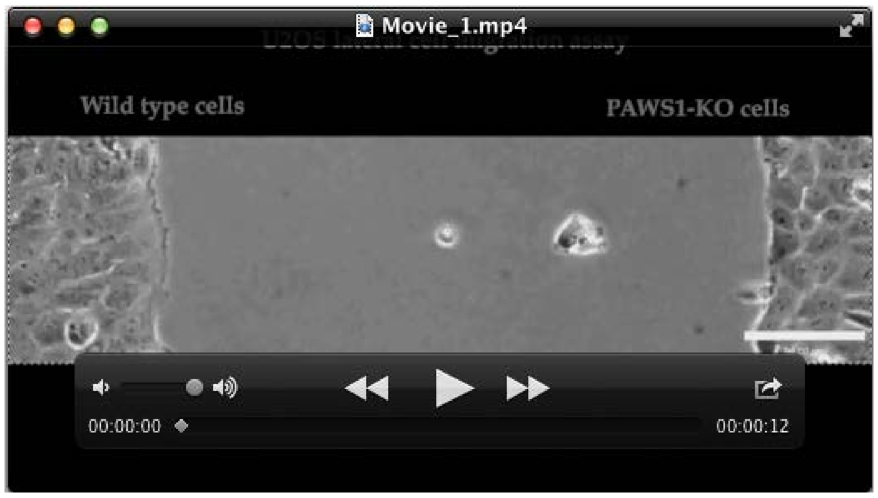

Movie 1. 2-dimensional lateral migration live-cell imaging using widefield microscopy. Live cell imaging of wild type U2OS cells on the left and PAWS1$^{-/-}$ U2OS cells on the right as indicated. Upon removal of the wound barrier, cells were allowed to migrate onto the gap for 16 hours. Images were acquired at 40X magnification every 5 minutes continuously.

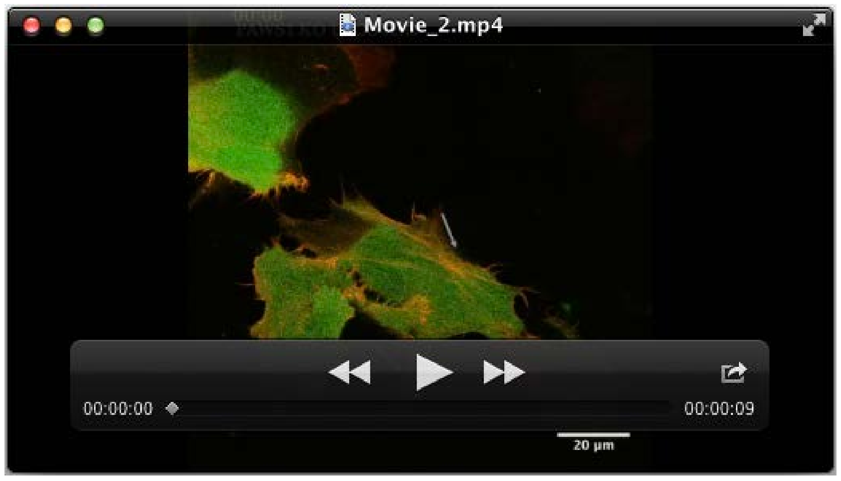

Movie 2. Live cell time-lapse fluorescence microscopy video of PAWS1$^{-/-}$ U2OS cells transfected with mApple-LifeAct (actin tracker) and GFP to visualize live cell actin dynamics. Images were acquired every 2 minutes over the course of a 25-minute time frame using a 60X magnification. The indicated areas exemplify lack of membrane ruffles. Scale bars are 20 μm.

Journal of Cell Science • Supplementary information

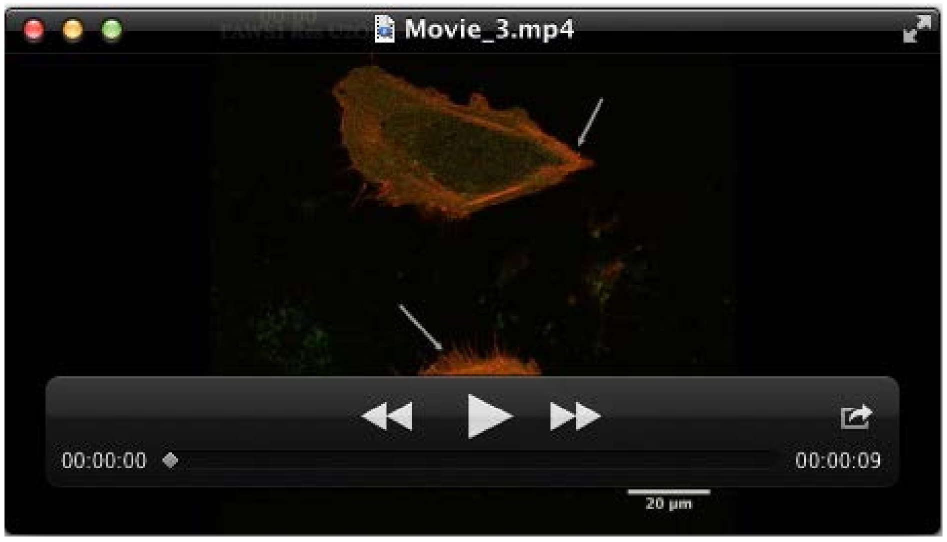

Movie 3. Live cell time-lapse fluorescence microscopy video of PAWS1$^{-/-}$ U2OS cells transfected with mApple-LifeAct (actin tracker) and GFP-PAWS1 to visualize live cell actin dynamics. Images were acquired every 2 minutes over the course of a 25-minute time frame using a 60X magnification. The indicated areas exemplify formation of membrane ruffles. Scale bars are 20 µm.

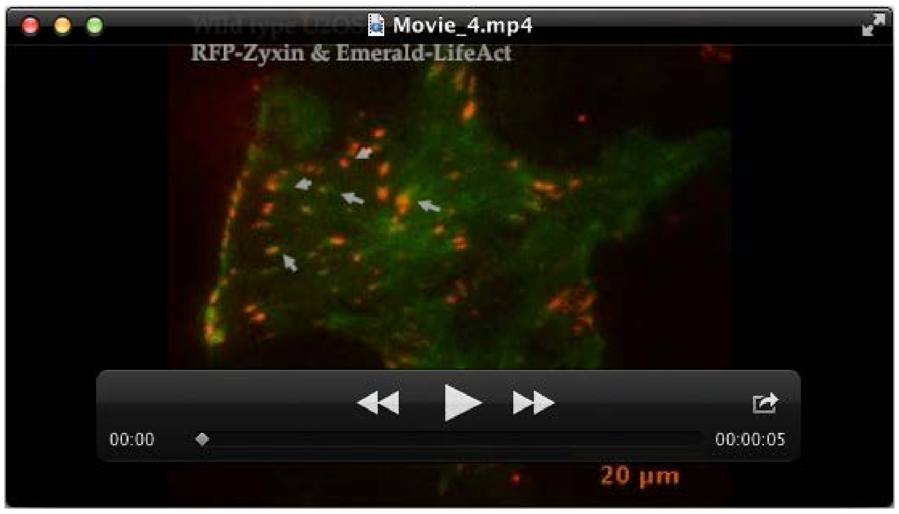

Movie 4. Analysis of focal adhesion dynamics in wild type U2OS cells using TIRF microscopy. Wild type U2OS cells were transfected with RFP-zyxin (red) and Emerald-LifeAct (green) to measure changes in focal adhesions over a time course of 25 minutes. Images were acquired continuously every 60 seconds for 25 minutes. Representative focal adhesion points are indicated by arrows. Scales bar indicates 20 µm.

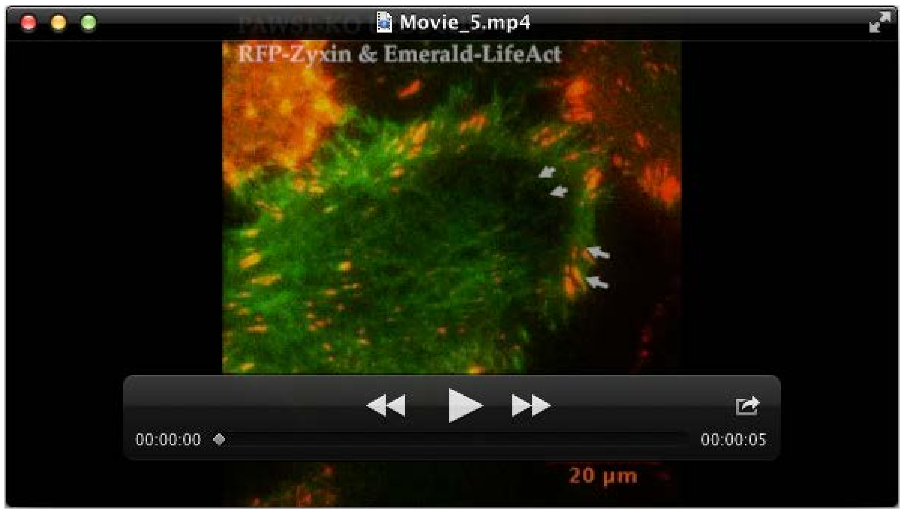

Movie 5. Analysis of focal adhesion dynamics in PAWS1$^{-/-}$ U2OS cells using TIRF microscopy. PAWS1$^{-/-}$ U2OS cells were transfected with RFP-zyxin (red) and Emerald-LifeAct (green) to measure changes in focal adhesions over a time course of 25 minutes. Images were acquired continuously every 60 seconds for 25 minutes. Representative focal adhesion points are indicated by arrows. Scales bar indicates 20 µm.

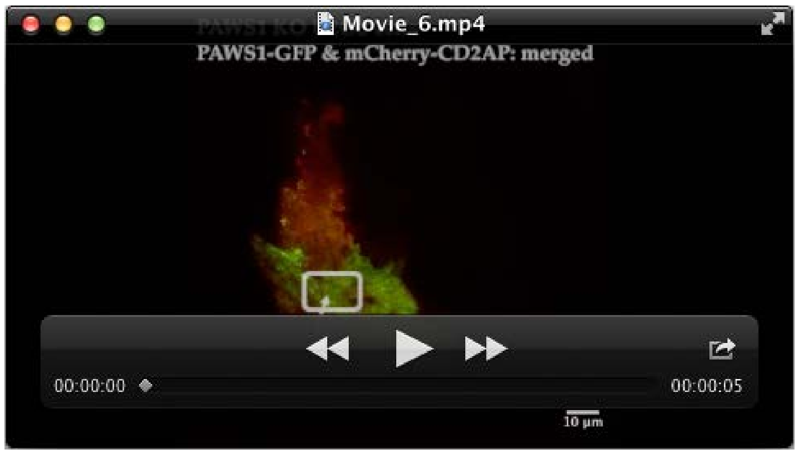

Movie 6. TIRF microscopy of PAWS1-GFP and mCherry CD2AP in PAWS1$^{-/-}$U2OS cells. Cells were transfected for 24 hours with PAWS1-GFP (green) and mCherry CD2AP (red) to measure changes in the co-localization distribution over a time course of 25 minutes. Merged channels shown. The movement of one co-localized punctate structure is boxed and indicated and highlights the dynamic nature of PAWS1 and CD2AP complexes in cells during the time course. Scale bars are 10 µm.

Journal of Cell Science • Supplementary information

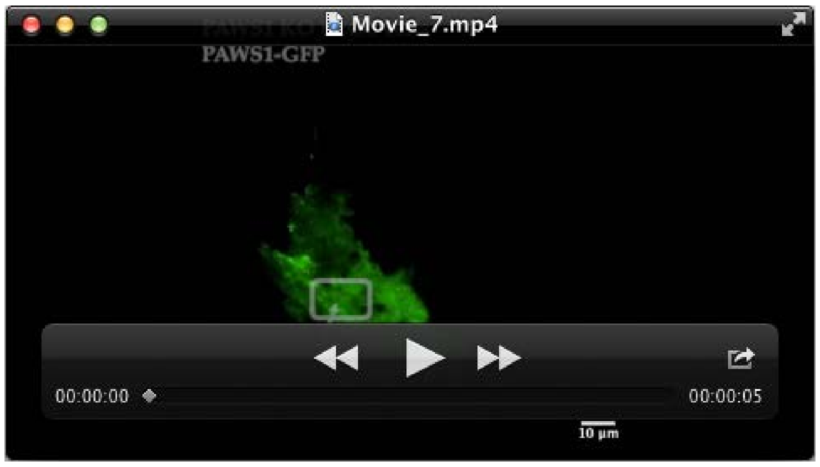

Movie 7. TIRF microscopy of PAWS1-GFP and mCherry CD2AP in PAWS1$^{-/-}$U2OS cells. Cells were transfected for 24 hours with PAWS1-GFP (green) and mCherry CD2AP (red) to measure changes in the co-localization distribution over a time course of 25 minutes. PAWS1-GFP (green) channel only is shown here. The movement of one co-localized punctate structure is boxed and indicated and highlights the dynamic nature of PAWS1 and CD2AP complexes in cells during the time course. Scale bars are 10 µm.

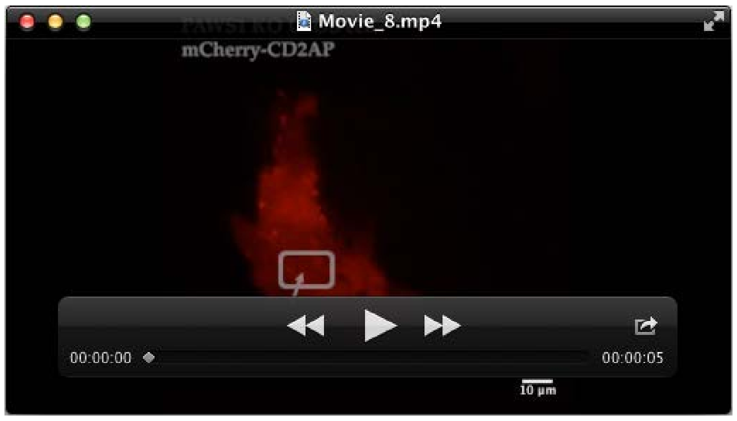

Movie 8. TIRF microscopy of PAWS1-GFP and mCherry CD2AP in PAWS1$^{-/-}$U2OS cells. Cells were transfected for 24 hours with PAWS1-GFP (green) and mCherry CD2AP (red) to measure changes in the co-localization distribution over a time course of 25 minutes. Only mCherry CD2AP (red) channel is shown here. The movement of one co-localized punctate structure is boxed and indicated and highlights the dynamic nature of PAWS1 and CD2AP complexes in cells during the time course. Scale bars are 10 µm.

Journal of Cell Science • Supplementary information
